# Hosting eSports events: the quality-response-behavioural intentions relationship of eSports fans

**DOI:** 10.3389/fspor.2025.1547097

**Published:** 2025-06-09

**Authors:** Luís Cerqueira, Tiago Ribeiro, Victor Manoel Cunha de Almeida

**Affiliations:** ^1^ Faculty of Human Kinetics, University of Lisbon, Oeiras, Portugal; ^2^ Research Center for Tourism, Sustainability and Wellbeing (CinTurs), University of Algarve, Faro, Portugal; ^3^ COPPEAD—The Graduate School of Business, Federal University of Rio de Janeiro, Rio de Janeiro, Brazil

**Keywords:** eSports events, physical environment, affective responses, behavioural intentions, eSports fans

## Abstract

**Introduction:**

The current study aims to explore how the physical environment of eSports events can influence fan affective responses and their future behavioural intentions. Following the Stimulus-Organism-Response (S-O-R) model, affective response (pleasure) is conceptualized as the organism (mediator) between the physical environment (stimulus) and behavioural intentions (response).

**Method:**

The dependent variables were revisit intention and word-of mouth, while demographic factors including age, education level, nationality, and event attendance to describe the sample and examine their potential influence. Data collection was carried out at a “Lisboa Games Week” event (*n* = 328) by using a self-administered questionnaire. A Confirmatory Factor Analysis analysed the psychometric properties of the constructs and a subsequent Structural Equation Modelling examined the substantive hypotheses tested.

**Results:**

Results indicate that the physical environment quality positively influences the affective responses of fans, which motivates them to follow eSports events. Furthermore, fans affectively attached to an eSports event are more intent on revisiting it and making word-of-mouth recommendations about it.

**Discussion:**

A high standard service quality is a critical issue for event managers, marketeers, and publishers due to its impact on the behavioural and affective value creation towards the event.

## 1 Introduction

The organized videogame competitions, also known as eSports, has grown and developed in the competitive events industry through an organizational model (1). These competitions include several stakeholders such as sponsors, sport organisations, and media (2), which provide high incomes and attendance levels. Such revenues have been visibly growing since 2018 (3), highlighting the impressive US$ 1.38 billion in 2022 and an expect growth to US$ 1.86 billion in 2025 (3). The total number of fans can be one of the arguments to justify such results considering the 532 million viewers across the world in 2022 (4).

In fact, eSports development has caught the attention of several researchers in the subject with a wide number of studies on sociology and sport management fields ranging from its classification as a sport (1, 5), its history (6), its future impacts on management (7), and fan motivations (8–10). However, despite its growth in sports and event management literature, few studies have focused on the physical environment at eSports events as a way to understand its effects on affective fan responses and behavioural intentions (11). At this point, service quality research has not been keeping up with the development of the eSports industry, opposing the events management literature which is wide and in-depth over this subject (2). Specifically, eSports events differ fundamentally from traditional sports in their spatial configuration, technological integration, and audience expectations (12, 13). Unlike traditional sporting arenas, eSports venues often incorporate hybridized environments where digital and physical elements converge to create a highly immersive experience (1). This hybridity necessitates a closer investigation into how specific physical environment cues, such as lighting and acoustics, can affect both emotional engagement and behavioural intentions among fans (10).

 Previous studies have highlighted the physical environment as one of the key factors belonging to the event service quality model [e.g. (14),]. A high-quality pattern may increase fan attendance and word-of-mouth in sports events (15) leading to a positive ticket revenue, impact on sales, attraction of sponsors and media, which in turn may add more financial gains and event value (16). According to Jang et al. (17), the physical environment research is relevant to predict fan affective and behavioural responses. This service quality construct has been widely researched in sport management with several models in different contexts [e.g., (17–22)]. However, its application within the context of eSports remains limited as there is a lack of empirical results validating its use in the literature on eSports events. As the industry continues to professionalize and invest in large-scale live events, understanding how the physical environment influences consumer behaviour becomes increasingly critical (23). Additionally, specific features to eSports such as virtual environments, online fan interactions, and unique event structures (24) have not been thoroughly examined in underlying their effect on fan affective and behavioural outcomes. This gap highlights the need for further research in order to explore the effects of eSports event quality on future fan behaviour, offering both theoretical insights and practical implications for event organisers and esports owners.

Understanding the role of physical environment quality in the eSports event on fan affective and behavioural responses is important for several reasons. First, eSports events have a different recreational culture than traditional events such as fans customizing their favourite characters [i.e., cosplay; (11)], different game time durations, and a heavy reliance on specific equipment to promote the event (2). Second, the event physical quality can awaken different reactions in fans (25), increasing or decreasing their emotional and affective responses towards the event. Through well-designed venues, engaging atmospheres, and high-quality production it is possible to enhance fan responses (22), whereas poor physical conditions can result in frustration or disappointment. Third, high standards of physical environment quality are critically relevant for both event organisers and fans due to their role on event social leverage (26). Based on these arguments, the current study aims to explore the effects of the physical environment on affective responses and behavioural intentions of fans towards an eSports event. Theoretically, this study advances the event management literature by empirically investigating to what extent fans are stimulated the physical environment to initiate a dynamic process of emotional responses with the eSports event. Using the Stimulus-Organism-Response (SOR) approach (27), the physical environment is represented as the stimulus that exerts influence over the fans' affective responses (organism), which subsequently triggers behavioural intentions as a response. Practically, this research will contribute to explore the fan's view of the event providing useful insights for managers and marketeers to review the physical event aspects and enhance future affective measures linked to fan interests. Understanding the correlation between Stimulus-Organism-Response could further engage fans with their esports-related activities and/or behaviours.

## 2 Literature review

### 2.1 Theoretical base

The SOR model is a useful theoretical lens that allows us to understand the influence from the fans' perceptions and emotions on their behavioural intentions (22, 28). This theory posits that a certain stimulus can cause an emotion on an organism and subsequently trigger a response or behaviour (27). The SOR model underscores the interrelationship among external stimuli, internal processes, and behavioural responses, offering a comprehensive understanding of how individuals perceive and engage with their environment (27). This model is powerful in predicting actual consumption behaviours such as attendance at events (17) and on-site spending (22). However, though previous studies have focused their research on different contexts [e.g., (17, 22, 25)], the relevance of the eSports physical environment and its impacts on future behaviours of WOM and purchase intention have rarely been examined in the sport management field. To this end, we argue that physical environment dimensions should be seen as a stimulus/antecedent (S), which induce fan affective responses (O), and subsequently generate future behavioural intentions (R).

Within the realm of eSports events, stimuli (S) can include physical elements such as colours, sounds, lighting, temperature, and other physical aspects of the environment (2). Organism (O) represents the individual and internal psychological processes. This can mean emotions, perceptions, or affective responses that influence how an individual interprets the stimulus. And the Response (R) is the observable behaviour of the individual to the stimulus provided in their environment (22). This may encompass emotional and behavioural responses such as the desire to return to the event, purchase intention, or spread favourable words about the event (29). In the eSports field, the SOR model helps us understand how the physical environment can influence fan perceptions, responses, and behavioural intentions (11), making it useful for marketers and managers to develop event planning and organization. This underscores the necessity for further research using the SOR framework to elucidate the complex interactions between environmental factors, fan behaviours, and the overall success of eSports events.

### 2.2 Esports events and their specificities

As organized videogame competitions, eSports feature their own events where players or teams are on the centre of the arena while the match is broadcast on giant screens to live and online audiences (23). These events integrate leisure stands where fans can go and try out videogames, hear lectures about new videogames, or play with friends (30). Similarly to traditional sports, eSports can be represented in multiple game genres such as First-Person-Shooter competitions (e.g., Counter-Strike 2 or Valorant), MOBA (i.e., multiplayer online battle arena such as LoL), RTS (i.e., real-time strategy such as Starcraft 2), and sport simulation games (e.g., FIFA 23) (31). These eSports games are organized towards live, online, and televised audiences, reflecting an exponential growth since their first editions until nowadays (1). For instance, the LoL World Championship in 2016 comprised a live audience of 20,000 fans along with more than 43 million online fans (1). Its exponential growth is seen in terms of quantity and quality in shaping coaches, referees, sponsors, fans, and media at live events as a part of its structure (10). The importance of these events is justified by its high profit, large audiences, and the attraction of fans attending these events (23).

Despite their structure being similar to traditional sport events, eSports events have different features and reactions. As noted by Zhu et al. (2), eSports events differ in several ways, including game duration (e.g., up to five hours compared to two to four hours in traditional sports), its dependence on equipment (e.g., viewed on screens and dependent on power sources and internet-connected devices), and its ludic culture (e.g., cosplay shows). Service quality patterns between eSports and traditional sport events can be different and vary according to the contextual setting (2). Although, eSports event literature gathers a wide range of studies from the consumption motivations of fans (8, 32), the influence of social environment (11), or the fans' brand image perception (33), there remains a need for a deeper understanding of the physical components that impact service quality supported by empirical findings (2). Moreover, eSports events differ from others as they require specific equipment such as consoles/computers, headsets, cables for a Local Area Connection (LAN), and gaming monitors for players and their fans (7). Also, commentators engage the crowd by narrating the match for both live and online viewers (11). For smaller or independent events, obtaining a license from the game publisher may also be necessary (23), making them distinct from traditional events. Esports further stand out due to their unique ludic culture, which includes fan cosplay shows (11) and various ancillary activities such as the opportunity to try out video games during the event or attend presentations from publishers (30).

### 2.3 Esports events: the physical environment quality

Physical environment is one of the dimensions of service quality with tangible characteristics and attributes that can influence fan perceptions (34), i.e., the physical or material side of a sports event. It plays a crucial role in creating an enjoyable experience for the fans (35), encompassing different attributes related to infrastructure, cleanliness, equipment, and accessibility (36). Its importance can be justified by the reliance on physical facility aspects where the eSports event is held such as giant screens, since without them fans cannot watch the team's live performance, or the internet connection systems so that the match can take place without interruptions (2). Through a reliable network infrastructure (2), sound and lighting, an incorporated digital design, eSports events might enhance fan experience, thus strengthening the affective and social bonds (37). These features distinguish the work tasks of event managers by enabling them to understand which physical environment attributes they should prioritize and recognize the specific needs of each consumer profile.

The physical environment quality at sports events has a wide assessment in sport management literature and includes a series of dimensions analysed [e.g. (20, 22),]. Wakefield et al. (19) have considered stadium accessibility, facility aesthetics, scoreboard quality, seating comfort, and layout accessibility as a part of a sports events physical environment structure. Yoshida and James (21) claim that the aesthetic quality represents the fans' perceptions over the service's visual attractiveness and ancillary products such as atmosphere, design, and themes (aesthetic) or frontline employees and facility functions (functional). Similarly, Ko et al. (20) include atmosphere, design, and signage in their study's survey (Scale of Event Quality in Spectator Sports) as physical environment dimensions in the baseball context. Physical dimensions have also been the subject of studies in other sports contexts, for instance football (22), baseball, basketball, and ice hockey (17). In the case of eSports, only Zhu et al. (2) have noted the features of this event type, highlighting the role of the physical environment as its structural model. However, despite their conceptual attempt to elucidate its role, there are no empirical studies that prove its effects in the eSports events context. By adding to the existing body of research, this study can help expand our understanding of the physical environment role, generating new insights for managers and inspiring further research on the topic. The theoretical rationale for the physical attributes conceptualisation and proposed hypotheses is outlined in the next sections.

### 2.4 Physical quality attributes that influence fan affective responses

There are four attributes of the physical environment quality that deserve particular attention: atmosphere, equipment, facility design, and accessibility. Atmosphere can be defined as the intangible characteristics and conditions within a sport facility that are capable of influencing the five human senses (38). Previous research has shown that the atmosphere of an event can impact fans' emotional responses in sports settings (22, 28, 39). A sport facility's intrinsic elements such as scent, light (28), cleanliness (28, 39), temperature (39), and sound (22) contribute to change the fans' attitudes toward the event. For instance, the opening show of the LoL World Championship 2022 included a set of lights and holograms creating the illusion of the performer being lifted by a mechanical hand (40). Based on the SOR framework, external environmental factors can elicit an emotional response to stimuli in the specific environment, influencing how individuals perceive and react to their surroundings (28). Considering that atmosphere conditions of the eSports arena can influence the fans' attitude, leading them to a different affective response during the event (2), the following hypothesis is proposed:

**H1**: An eSports event atmosphere positively influences fan affective responses.

Equipment refers to the use of devices and instruments that ease and maximize spectator consumption (14). Several items of this dimension have been described as positive influencers on fan affective responses such as scoreboard quality, seat quality (17), or the electronic equipment quality (39). By using a reliable network infrastructure (e.g., high-speed internet connection), adequate screens, and backup systems (e.g., high quality screens, power generators), it is possible to prevent disruptions during the event (2). An eSports event largely depends on this physical dimension (37), which can significantly influence the experience for both players and fans attending the event. Taking into in account that it is possible for equipment quality to influence fan perceptions and provide different affective responses during the event, the second hypothesis is formulated:

**H2**: An eSports event equipment positively influences fan affective responses.

Facility design is related to the architecture and decoration of the facility such as its structure colour (17). This attribute is highly valuable in service quality since fans make their first impression of service delivered based on the visual aspect of the facility (19). It should be noted that elements such as decoration, architecture (21), stadium, and entrance space (41) can also affect this perception. In fact, fans spend a lot of time looking to the facility design elements, which can further influence their reactions over the event quality (19). For instance, in the LoL World Championship 2021, a decoration similar to Netflix's show “Arcane” was produced to impact millions of fans through facility visual design (42). As such, it is expected that a high design standard allows fans to interact during the event, increasing their identity and connection with the community (24). Considering that this dimension is a crucial conceptual measure of the physical environment (2, 37), it is anticipated to impact the affective responses of fans attending an eSports event, thus the following hypothesis is presented:

**H3**: An eSports event facility design positively influences fan affective responses.

Accessibility is the ease that fans can reach their intended destinations in a sports facility (19). Elements such as the ease to enter and leave the event or to reach restrooms have a high importance in sports events since they can influence the fans' experience and their perceived quality (41). Although this dimension has not been highlighted in recent studies [e.g., (2, 37)], previous research has denoted that accessibility has a positive impact on fan affective reactions [e.g., (17, 21)]. Some attributes such as the signage (19) or event layout (17) can influence fans' emotions and responses. Moreover, in 2022, there were 560 million individuals with disabilities actively participating in eSports (43), underscoring the need for ensuring adequate safety measures for everyone involved. At live eSports events, it is crucial to implement accessible features such as ramps, wide aisles, and appropriate seating arrangements to accommodate all types of fans, including those with disabilities. Considering that accessibility inside an eSports arena is crucial to support crowd management and information, we decided to include the accessibility dimension in our structural model. Therefore, the following hypothesis is suggested:

**H4**: An eSports event accessibility positively influences fan affective responses.

### 2.5 Fan affective responses and their behavioural intentions

These affective emotions or reactions refer to an emotional state that is developed in individuals caused by their perception of the surrounding environment (44). Following Mehrabian and Russell (27), there are three emotional states between the stimulus and consequent responses—pleasure, excitement, and dominance. Pleasure is defined as the sensation of feeling good (happy about a situation), excitement reveals the degree to which an individual feels stimulated or active in a situation, while dominance is seen as the individual's feeling of influence and control (45). These responses can act as an organism, mediating the process between physical environment (antecedents) and behavioural intentions (consequents) (22). Jang et al. (11) noted the affective responses as an organism can influence fan revisit intention in the eSports field, while Ryu and Jang (45) highlighted the emotional state of pleasure as a multidimensional response on costumer behavioural intentions. While these studies have noted that sports events physical and social environment (22), as also an eSports social environment (11), can influence the future behavioural intentions of fans, it is important to explore how an eSports physical environment can influence fan emotions and future behavioural intentions as it is a new event context, which can bring new conclusions to the SOR model. The Jang et al. (17) study is an example that brings a variation of results across different sports events contexts that resulted in different fan perceptions, emotions, and behavioural intentions.

Esports events can generate different affective responses from fans, leading to multiple individual behaviours emerging (27). Biscaia (16) described these behaviours as responses to a satisfactory experience on the part of the viewer, which is preceded by the perception of several factors, including the quality of the service. Fan behavioural intentions are reflected in their interest in the event, its durability (19), the intention to revisit it (17) or to recommend it to others (22). In an eSports context, Jang et al. (11) highlighted that such behaviours act as a response element (e.g., intention to revisit), underlying in a stimulus originating in an organism. These authors have noted that watching more hours of the game, speaking positively about the event, or a greater commitment to the game watched are behavioural intentions shown by eSports fans. In the present study we defined two behavioural intentions by describing revisit intention as the intention to attend future events and word-of-mouth recommendation as a way of suggesting the event to others.

When considering that an individual's affective responses can lead to approaching or distancing behaviours in the environment experienced (27), it becomes essential to understand how various environmental stimuli influence these emotional reactions. Positive affective responses are likely to encourage fans to engage more fully with the event, leading to behaviours such as staying longer, revisiting the event, or interacting with others. By eliciting such emotions through eSports events, managers and marketeers can promote strong emotional connections with fans, increasing fan loyalty and driving purchase intentions (28). Considering that high emotional levels on digital event can positively influence their consumption behaviour, including intention to revisit the event (11) and their intention to spread word-of-mouth (46), the following hypotheses are proposed:

**H5**: Esports fans' affective responses positively influence their intention to revisit the event.

**H6**: Esports fans' affective responses positively influence their word-of-mouth intention.

## 3 Methods

### 3.1 Contextual setting

This case study is focused on the “Lisboa Games Week” (LGW) held in Lisbon, Portugal between November 17 and 20, 2022. The event featured the finals of the Worten Games Ring (WGR)—Portuguese League of Legends League and the WGR—Valorant Legion VCE 2022 Elite Championship competitions, which took place on November 19 and 20, 2022. These organized events had a maximum prize of 50,000 euros and 20,000 euros, respectively (47). The event was overseen by Feira Internacional de Lisboa (FIL) and Inygon with distinct organizational roles and responsibilities assigned across multiple functional areas.

FIL is a Portuguese organization established in 1957 that includes a 100,000 square-meter space in Parque das Nações, Lisbon (48). Its mission is to organize and promote national and international fairs and exhibitions, facilitate experiences, and foster innovation and design for economic stakeholders involved in the fairs (60). Inygon is a Portuguese company founded in 2015 that delivers organization and transmission services of eSports competitions besides its additional services around event organization and software development. They are currently responsible for managing and organizing two of the largest Portuguese eSports competitions—the Portuguese League of LoL and Valorant Challengers Portugal: Tempest. It should also be noted that they are responsible for broadcasting eSports competitions in Portugal, including the LoL World Championship (49).

### 3.2 Sample and data procedures

This is a quantitative, cross-sectional study. A non-representative sample of fans who attended the LGW—WGR (*n* *=* 328) and all participants voluntarily agreed to participate and signed the informed consent form. The questionnaires were collected during the event (LGW—WGR) for two consecutive days (i.e., November 19 and 20, 2022) within the facilities hosting the event (i.e., FIL Lisbon). A team of 5 junior researchers from a local university and two senior supervisors collected the data in person at the event location. Each junior researcher was assigned by the supervisor to administer the questionnaires in a specific area of the event. The researchers approached potential participants, explaining the purpose of the study, its benefits, and risks during participation. The following criteria were considered for selecting participants: (i) fans who were present at the event location, (ii) fans who had attended at least one eSports competition of the event, (iii) fans fluent in Portuguese, and (iv) fans aged 18 years or older (i.e., adults). Respondents could only complete the survey if all four conditions were met. All participants voluntarily agreed to participate and signed an informed consent form.

A total of 328 questionnaires were collected. Data were examined and questionnaires that were not completed were excluded from the sample. After data screening, 323 complete responses were considered useful for analysis. More than two-thirds of participants were male (70.7%) aged between 18 and 55 years, predominantly in the 18–25 age group (60.7%). Specifically, 50.5% of participants (global sample) reported that their highest level of education was college, almost 46.3% had completed high school, followed by 2.7% of participants with a middle or elementary school level of education. Most respondents were Portuguese (94.5%) with 5.5% of other nationalities but residing in Portugal. Regarding attendance at the event, the results indicate that 98.5% of participants watched at least one eSports game during the event with half of the sample revealing that they had watched LoL (50.6%) and the other half Valorant (47.9%). Table 1 presents the demographic characteristics of the fans.

**Table 1:** Demographic characteristics and MANOVA results.

Variable	Item	Total Sample (*n*/%*)*	PQ		AR		BI	
			*F*	*p*	*F*	*p*	*F*	*p*
Gender	Men	232/70.7	.042	.838ª	.686	.408ª	.389	.634ª
	Women	96/29.3						
Age	18–25	199/60.7	.519	.670ª	.481	.696ª	.397	.755ª
	26–35	108/32.9						
	36–45	15/4.6						
	46–55	6/1.8						
	Mean age (SD)	22.7 (.672)						
Education level	Elementary School	1/0.3	1.22	.296ª	.794	.555ª	1.33	.250ª
	Middle School	9/2.7						
	High School	152/46.3						
	Academic Degree	166/50.5						
	* (Bachelor)*	133/40.5						
	* (Master)*	29/8.8						
	* (PhD)*	4/1.2						
Nationality	Portuguese	310/94.5	1.14	.338ª	.335	.854ª	.363	.835ª
	Other	18/5.5						
Event attendance	Yes	323/98.5	.535	.465ª	.154	.595ª	.019	.890ª
	No	5/1.5						
Game genre	Legue of Legends	166/50.6	.566	.453ª	.852	.357ª	.165	.685ª
	Valorant	157/47.9						

ª No statistically significant differences were found.

PQ, physical quality; AF, affective responses; BI, behavioural intentions.

Source: Authors own creation.

### 3.3 Measures

This study's questionnaire consisted of a total of 27 items. The first section of the questionnaire collected sociodemographic information (e.g., age, sex, nationality, and education level), while the second part examined the physical environment quality in relation to the psychometric measures analysed (i.e., atmosphere, equipment, facility design, and accessibility), affective responses (i.e., happy or unhappy, satisfied or unsatisfied, and delighted or disappointed), and viewers' behavioural intentions (word-of-mouth and revisit intention).

A guiding question invited respondents to evaluate the items according to their level of agreement and two initial filter questions were included related to whether the participant had watched the event or not and what type of eSports he/she watched during the event. Respondents who answered “No” or “I don't know” to any of these filter questions were excluded from this study. Then, all participants were invited to evaluate the following dimensions:

*Atmosphere.* Based on aspects of the facility that affect the human senses (sight, touch, hearing, smell, and taste), a 5-item scale was adapted from Hightower et al. (28).
*Equipment.* Considering the tangible equipment relevant for the normal running of the event (e.g., size of giant screens or the sound system), a 5-item scale was adapted from Wakefield et al. (19) and Wakefield and Blodgett (39).
*Facility Design.* Taking in account the design, architecture, and decoration of the facility such as the colours used or the attractive architecture (17), a 4-item scale was adapted from Yoshida and James (21).
*Accessibility.* Referring to the ease with which all participants could navigate and fully engage with the event, regardless of their physical abilities or limitations (36), a 4-item scale was adapted from the Yoshida and James' (21) and Childress and Crompton' (50) scales.
*Affective Responses.* The affective response measured in this study was pleasure, defined as the degree of happiness of an individual (27). A 3-item scale was adapted from Jang et al. (17).
*Revisit Intention.* This refers to the fans' intention to return to or attend the event again in the future (17). A 3-item scale was adapted from Jang et al. (17) and Tsuji et al. (51).
*Word-of-mouth.* This refers to the fans' willingness to share positive feedback or recommend the event (16). A 3-item scale was adapted from Uhrich and Benkenstein (22).

All items used in this study were translated from English into Portuguese and back translated into English to ensure accuracy between the original scales, the translated version, and the accuracy of the wording given the cultural context (52). The content validity process (53) was ensured by two academics with experience in managing sports events and a professional from the event itself (i.e., Inygon). The researchers were instructed to raise any doubts while filling out the scale and the content analysis of the items. After this stage, suggestions to change the wording were made for 7 items to improve the understanding of each of the statements. All items were formulated based on positive statements and were mixed within each section. The questionnaire included seven-point Likert scales (from 1 = “Strongly disagree” to 7 = “Strongly agree”) and the items can be found in the Supplementary Appendix.

### 3.4 Data analysis

Descriptive statistics were calculated using SPSS 26.0 followed by data analysis using AMOS 26.0. A Confirmatory Factor Analysis (CFA) was performed on the model proposed to ensure the psychometric properties of the measurement model. Then, the substantive hypotheses were tested using Structural Equation Models (SEM), which simultaneously uses a series of separate and independent multiple regression equations (54). The ratio of chi-square (*χ*^2^) to their degrees of freedom, Tucker–Lewis Index (TLI), comparative fit index (CFI), goodness-of-fit index (GFI), and root mean square error of approximation (RMSEA) were the fit indices used in this study (55). Convergent validity was assessed in terms of factor loadings through the average variance extracted (AVE), while discriminant validity was assessed by comparing squared correlations between the constructs (56). Regarding internal consistency, Cronbach's alpha and composite reliability were measured to assess the survey measures' reliability. Subsequently, a series of MANOVAs were conducted using IBM SPSS 26.0 to examine the relationships between socio-demographic variables and dependent variables (see Table 1).

## 4 Results

### 4.1 Descriptive statistics and assumption tests

Table 2 presents the descriptive statistics. All skewness values (lower than −1.899) and kurtosis (lower than 4.720) indicated a normal distribution of the data. Further analysis through correlation (<0.59) and variance inflation factor (ranging from 1.23 to 2.03) showed no severe concerns with multicollinearity (57). The data were positively skewed with mean scores significantly above three (3.5 values), which represents the midpoint of the 7-point Likert scale items, for all factors. The highest average scores were evidenced by the perception of the atmosphere and equipment (*M*_atmosphere_* =* 5.67 and *M*_equipment_* =* 6.37) followed by design (*M* *=* 5.43) and accessibility (*M* *=* 4.77), revealing the high standard of quality of the physical environment. The results also indicated that the intention to revisit the event had a higher mean score (*M* *=* 5.38; *SD* *=* 1.48) than the word-of-mouth recommendation (*M* *=* 4.98; *SD* *=* 1.66), showing a value higher than 3.5 (average value). Furthermore, all variables were positively and significantly intercorrelated.

**Table 2:** Correlation matrix, AVE values, and square correlations between constructs.

Constructs	M(SD)	Correlation matrix (*n* *=* 328)							Factor weights	Z-Value	*α*	CR	AVE
		1	2	3	4	5	6	7					
1. Atmosphere	5.67 (.95)	**.** ***51***							.545–.816	9.45–15.24	.72	.73	.50
2. Equipment	6.37 (.77)	.25	**.** ***62***						.772–.797	13.09–13.21	.82	.83	.62
3. Facility Design	5.43 (1.12)	.30	.15	**.** ***61***					.718–.896	13.10–18.39	.84	.85	.65
4. Accessibility	4.77 (1.31)	.26	.11	.46	**.** ***62***				.559–.882	10.45–18.37	.82	.83	.62
5. Affective Responses	5.63 (1.11)	.22	.10	.28	.20	**.** ***72***			.819–.900	16.98–18.50	.88	.89	.72
6. Revisit Intention	5.38 (1.48)	.13	.03	.23	.21	.35	***.77***		.743–.947	17.93–30.08	.90	.91	.77
7. Word-of-mouth	4.98 (1.66)	.17	.04	.26	.30	.39	.59	**.68**	.796–.844	16.34–17.91	.86	.87	.68

None of the correlations failed the discriminant validity test.

Bold values represent AVE values.

M, mean; SD, standard deviation; α, cronbach alpha; CR, composite reliability; AVE, average variance extracted. The diagonal values refer to the AVE.

Source: Authors own creation.

Separate MANOVA analyses revealed that fan perceptions do not vary significantly between independent variables across the dimensions analysed. This means that factors such as gender, age, education level, and event attendance do not significantly influence how fans perceive the overall dimensions. In other words, the fan's perceptions remain consistent across these demographic and behavioural categories, indicating that these variables did not play a decisive role in shaping fan attitudes in the eSports event context. This result suggests a general uniformity in fan perceptions regardless of demographic characteristics or individual experiences.

### 4.2 Model assessment

The CFA results showed that the factor loading of 6 items did not exceed the cutoff point of 0.50 (57) and, consequently, the items were eliminated from the scale. The overall fit indices indicate that the measurement model proposed provides a good fit to the data [*χ*^2^(168) *=* 411.48 (*p* *<* *.*01), *χ*^2^/*gl* = 2.44, CFI = .94, GFI *=* *.* 90, NFI = .91, TLI = .93, RMSEA = .06]. The CFI, NFI, and TLI values exceeded the recommended cutoff of 0.90, while the RMSEA value was favourable considering the 0.08 threshold (55). As shown in Table 2, all items presented acceptable factor loadings, ranging from 0.54 to 0.94. *Z* values ranged from 9.45 to 30.08, suggesting that the items accurately captured their respective factors (44). Furthermore, Cronbach's alpha and composite reliability values (*α* and *CR*) of all constructs exceeded the recommended threshold of 0.70 (values greater than 0.72), providing support for the internal consistency of these constructs (58).

Each factor presented reliable and valid psychometric properties (convergent and discriminant). AVE values ranged from 0.50 (atmosphere) to 0.77 (intention to search the event), exceeding the recommended limit of 0.50 and providing evidence of convergent validity (56). Furthermore, evidence of discriminant validity was accepted since the correlation coefficients were lower than the suggested criterion of 0.85 (61) and none of the squared correlations exceeded the AVE values for each associated factor (56). The correlation matrix for the constructs is presented in Table 2.

**Figure 1:**
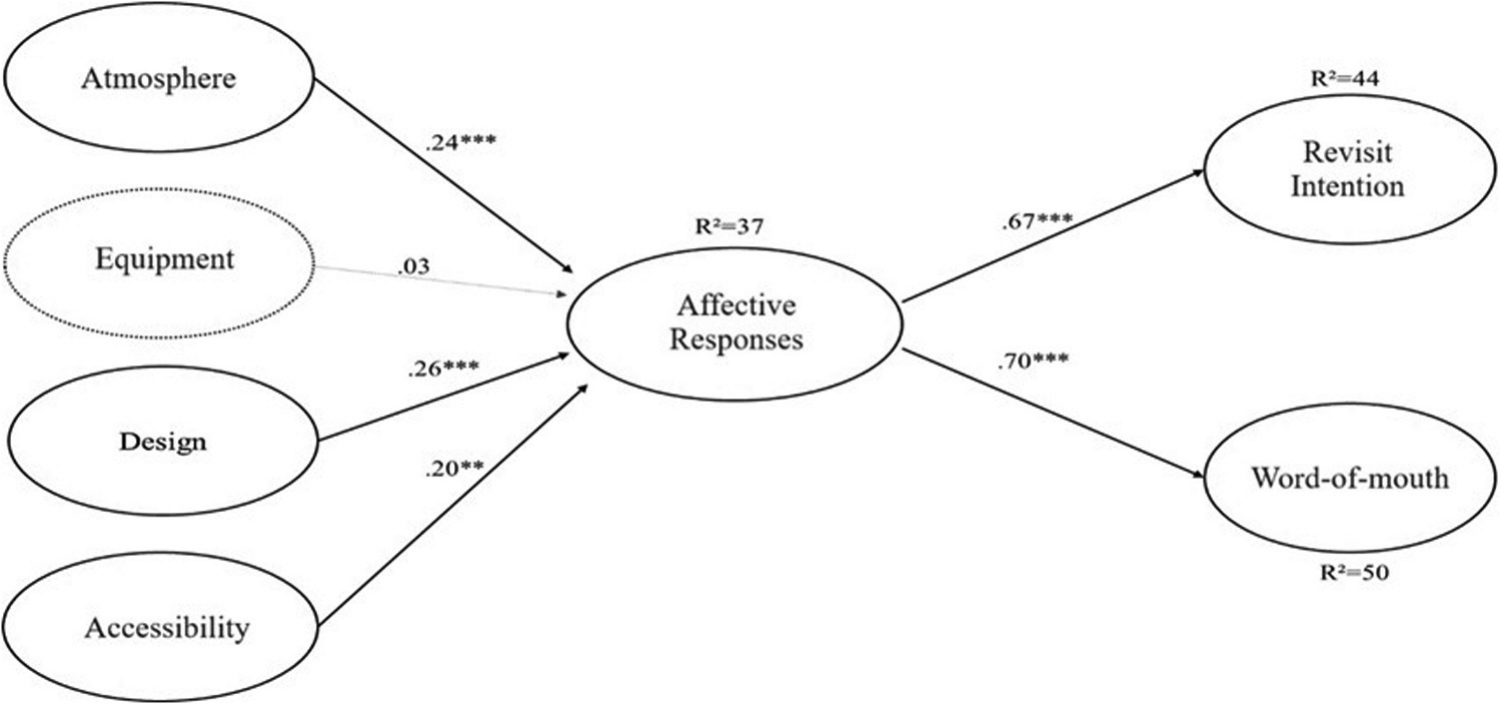
Standard estimates of structural relationships between constructs. Notes: ***p < .01; **p < .05. Source: Authors own creation.

The overall assessment of the structural model showed an acceptable fit [*χ*^2^(176) = 522.75 (*p* *<* *.*01), *χ*^2^*/gl* *=* 2.97, CFI = .92, GFI = .88, NFI = .88, TLI = .90, RMSEA = .07]. Figure 1 reports the structural relationships in the model, highlighting that all hypotheses were supported except for H2. The constructs of atmosphere (*β* = 0.24, *p* < 0.01), design (*β* *=* 0.26, *p* *<* 0.01), and accessibility (*β* *=* 0.20, *p* *<* 0.05) showed a positive and significant relation with the affective responses of eSports fans, thus supporting H1, H3, and H4. In turn, the path coefficient from affective responses to intention to revisit (*β* *=* 0.67, *p* *<* 0.01) and positive word-of-mouth (*β* *=* 0.70, *p* *<* 0.01) was also positive and significant, therefore, H5 and H6 were supported. The constructs of the physical environment quality were responsible for approximately 37% of the “affective responses' variation (*R*^2^ *=* 0.37) and approximately 44% of the “intention to revisit' variation (*R*^2^ *=* 0.44) and 50% of the “word-of-mouth' variation (*R*^2^ *=* 0.50). The coefficients path for each model are illustrated in Table 3, indicating the results of the structural model.

**Table 3:** Results of SEM analysis and indirect effects.

Hypothesis/path	SOR Model for eSports		
	Supported?	*β*	*t*-value
Direct effects			
*H1* Atmosphere → Affective responses	Yes	.242***	2.91
*H2* Equipment → Affective responses	No	.030^n.s.^	.450
*H3* Facility Design → Affective responses	Yes	.258***	3.06
*H4* Accessibility → Affective responses	Yes	.197**	2.41
*H5* Affective responses → Revisit Intention	Yes	.666***	12.25
*H6* Affective responses → Word of mouth	Yes	.704***	11.51
	r	[95% CI]	*p*-value
Indirect effects			
AT → RI	.282**	[0.03–0.60]	.032
AT → WOM	.317**	[0.02–0.65]	.038
EQ → RI	.038^n.s.^	[−0.21 to 0.27]	.758
EQ → WOM	.043^n.s.^	[−0.19 to 0.30]	.754
FD → RI	.374**	[0.16–0.67]	.002
FD → WOM	.421**	[0.14–0.76]	.002
AC → RI	.173^n.s.^	[−0.03 to 0.40]	.094
AC → WOM	.194^n.s.^	[−0.03 to 0.46]	.088

*** *p* < 0.001.

** *p* < 0.05.

n.s. = not significant; 95% CI = 95%.

β, direct effect.

Bias-corrected confidence intervals.

Source: Authors own creation.

The bootstrapping method and bias-corrected (BC) 95% confidence intervals were applied to estimate the paths for indirect effects in the model. The equipment and accessibility variables did not have a significant or indirect impact on word-of-mouth or the intention to revisit the event (see Table 3). Both the atmosphere [*r*_revisit_ = .28, *p* *<* .05, 95% CI = [0.03, 0.60]; *r*_wom_ = .32, *p* *<* .05, 95% CI *=* [0.02, 0.65]] and the facility design [*r*_revisit_ = .37, *p* *<* .05, 95% CI *=* [0.16, 0.67]; *r*_wom_ = .42, *p* *<* .05, 95% CI *=* [0.14, 0.76]] showed a positive indirect effect on behavioural intentions (see Table 3). Affective responses were found to partially mediate the relationships between both facility design and behavioural intentions, and atmosphere and behavioural intentions. These findings suggest that both facility design and atmosphere in esports can influence fans' future behavioural intentions through the additional affective bonds of fans. Table 3 presents the indirect effects in detail.

## 5 Discussion and implications

The purpose of this study was to examine the influence of the physical environment on fan affective responses and behavioural intentions at eSports events. The findings revealed that attributes of the physical environment positively affect the emotional responses of fans. Specifically factors such as accessibility, facility design, and atmosphere significantly shaped fan quality perceptions, eliciting positive emotions that in turn contributed to their future behavioural intentions. These findings support the hypotheses proposed and offer practical implications for the eSports event industry, highlighting the importance of optimizing physical environment attributes to enhance fan experience and their future behaviour.

### 5.1 Theoretical implications

The “accessibility' dimension was included in the conceptualization of the model not only because of its importance in previous studies (17, 21, 36), but also because of the number of people with disabilities who actively participate in eSports. This factor has evidenced added value to the conceptual model, highlighting a positive and significant relationship with the affective responses of eSports fans (see Figure 1).

The structural relationship between the atmosphere and affective responses of fans was also confirmed (H1). Considering that this eSports event was carried out in a closed space, the ability to manipulate these aspects is greater, similar to what was mentioned in relation to sports traditional events (36). According to Wakefield and Blodgett (36), the atmosphere in a closed environment is easier to control, which may enrich the fan's experience. For instance, atmosphere elements such as lighting, sound, temperature, and seating arrangements can be strategically managed to create a distinctive and immersive environment (37). As a result, if an eSports spectator is involved in an event whose atmosphere has quality and control, positive affective responses are generated, triggering subsequent behavioural intentions, which is in line with the SOR theory (27).

Facility design obtained the highest coefficient path in influencing fan affective responses. Its high effect on affective responses is also confirmed by the model of Zhu et al. (2). Following the event management literature, this dimension is often the first impression formed by fans regarding the service provided (19). The fact that fans spend a lot of time at the event (2), observing the aesthetic elements of the eSports arena can influence their perceptions of aesthetic quality (19), thus contributing to explaining their affective responses. This specific event, the LGW—WGR, took place from 3:00 pm to after 8:00 pm on both days of the event, which may explain this effect. Following the SOR theory, the facility design assumes a stimulating role, generating affective responses in the organism (fan) and triggering a response (revisit intention and word-of-mouth). Our findings corroborate previous literature on atmosphere (22, 28, 39) as well as facility design and accessibility in the context of traditional sports (17, 21), showing that well-designed and accessible facilities significantly enhance fan emotional answers and future behaviours. This positive emotional state further contributes to their likelihood of returning to future events and recommending the experience to others, emphasizing the importance of these physical environment dimensions in shaping fan attitudes and intentions.

In contrast, the equipment dimension has showed a non-significant effect in influencing the affective responses of fans, contrary to what is indicated by previous studies (17, 39). However, this dimension is referred to as preponderant in an eSports event (2) since a failure in equipment (e.g., internet connection or hardware) can affect viewing of the game and the spectator's experience, and consequently influence the fans' perceptions. An explanation for this finding could be linked to the high quality of the equipment, which fulfils fans' expectations, but does not exceed them. Although the design quality is of a high standard, it merely meets the viewers' basic expectations, thus failing to elicit significant emotional responses (25). As a result, when a service merely meets consumers' minimal expectations, it may not be sufficient to elicit emotional responses, which aligns with the findings obtained. Another additional explanation for this result may be related to the fact that this event took place in an open space. There was no physical separation between the different brands, sponsors, and activities that took place independently of the event, which may have resulted in an additional distraction for fans. Furthermore, it is important to note that this equipment (screens, sound system, seats) is described as the use of devices and instruments that facilitate and maximize spectator consumption (14), which take on a secondary role in the context of sport. However, despite the equipment dimension not yielding significant results concerning fan affective responses, it is essential that it be included and examined in future studies, potentially for replacing scale items.

The results also indicate that affective responses have a positive and significant effect on behavioural intentions, particularly the intention to revisit the event and word-of-mouth recommendations (see Table 3). One of the reasons given for the importance of the physical environment in sports events was the increase in the number of stadium fans and consequent financial gains (18). Thus, the intention to attend the arena again (intention to revisit) and the recommendation of the event to third parties (positive word-of-mouth) may become relevant for retaining fans at a new event and the visit of new fans (59). Similar to the effects of the physical environment constructs of sports traditional events, the physical environment constructs of eSports events can also influence fan affective responses and future behavioural intentions. This relationship may result in an increased number of fans at future events and associated financial gains, which is aligned with previous literature on behavioural intentions in the context of eSports [e.g., (11)]. Consequently, the fact that affective responses act as an organism between the constructs of physical environment (stimulus) and behavioural intentions (response) corroborates the SOR theory (27).

The affective responses played a mediating role in the relationship between the physical environment and the behavioural intentions of eSports fans. This partial mediation suggests that while the physical environment directly contributes to fans' revisit intentions and word-of-mouth, its effects are also transmitted through fans' emotional states. Our results revealed that atmosphere and accessibility showed significant indirect effects on behavioural intentions via affective responses, offering additional empirical support for this pathway. By improving such services, the quality pattern may be increased, consequently encouraging an affective reaction and therefore positively influencing behavioural intentions. Although some previous studies have assumed a direct relationship between service quality and behavioural outcomes [e.g., (59)], our findings support a better understanding of emotional reactions as a key variable. By applying the S-O-R framework in the eSports context, this study extends its relevance to digital-native event experiences and emphasizes the need for organizers to consider both functional and emotional dimensions of service delivery.

This study advances on the sport management literature through three key contributions: (1) the conceptualization of a model based on Zhu et al. (2) with the inclusion of the accessibility factor; (2) the presentation of empirical findings within the Quality-Response-Behavioural intentions chain; and (3) the application of the SOR theory within an eSports small-event context. By investigating SOR theory in relation to service quality, this research enhances our understanding of how environmental stimuli and emotional responses interact to influence fan behaviours in service settings. Following our findings, we can argue that when eSports fans perceive high physical environment patterns, positive affective responses can occur, encouraging them to spread favourable word and revisit the event. To this end, this study contributes to advance event management literature through its application in an unexplored social context, considering the quality-response-behavioural intentions chain to explain how the physical environment can trigger different affective responses that impact future behavioural intentions.

### 5.2 Practical implications

The social atmosphere, facility design, and accessibility should be considered by eSports managers and event marketeers as key aspects to improve the fan's attitudes and behaviours. It is important to have attractive architecture at the arena and the width of the entrances and accesses must be considered to allow easy circulation of the public (e.g., crowd management). As mentioned in the study by Wakefield et al. (19), these stimuli are extremely important as fans form their first perception of the event by observing the facility design. Considering that it is one of the fans' most observed elements, it is essential to invest financial resources in improving the physical and virtual design of the installation.

Secondly, this study highlights a significant relationship between atmosphere and fan affective responses, which is of particular interest to eSports managers and marketers due to the sensory stimuli experienced by fans. For instance, the quality and innovation of both general and stage lighting are areas that warrant attention, especially considering advancements in lighting and holographic technology, as evidenced during the LoL World Championship 2022. Additionally, exploring the musical preferences of fans could provide valuable insights for incorporating their favoured musical styles throughout the event.

According to the results, the quality of the venue accessibility has a positive and significant relationship with affective responses from fans. In this sense, it is important to invest in signage that is visible and with precise and continuous directions (e.g., placing signs that indicate these changes), so that the spectator can reach the desired location easily and quickly. It should be added that for disabled people, accessibility requires due care so that there are no obstacles to the movement and viewing of these fans. The construction of ramps with the appropriate slope, elevators in stairwell areas, the placement of seats with a good view, or the placement of a sign language interpreter (establishing a connection with the commentators' words) are just a few examples that can improve the experience of these fans.

Finally, while the equipment dimension did not yield significant results in influencing fan affective responses, it should not be overlooked. A malfunctioning screen or sound system can disrupt the ongoing match and negatively impact the spectator experience. To mitigate this risk, it is essential to enhance quality and innovation in this particular dimension. For instance, the installation of larger screens, surround sound systems for improved spectator immersion, and innovations in game viewing such as 3D transmission could significantly enhance the overall fan experience.

## 6 Limitations and further research

This study has several limitations that may present new opportunities for future research on this topic. First, the questionnaire was administered in person without any barriers separating the event space from the broader Lisboa Games Week venue. This area featured various attractions surrounding the eSports arena, including IT equipment brands and virtual reality simulators, which could influence fan perceptions of event quality and, consequently, their responses to the surveys (36). Future research should explore alternative sampling strategies and consider events held in enclosed spaces, minimizing the impact of unrelated variables.

Second, the questionnaires were administered over two days where 2 different eSports events took place. Results were collected regarding viewers of the LoL game and other viewers of the game Valorant. Although the physical environment presented was the same on both days, it must be considered that these are fans of different games (MOBA and FPS). This limitation can translate into different spectator perceptions regarding the quality of the physical environment of the event. In further research it would be important to explore the physical environment of an event that promotes only the competition of one type of game or carry out the appropriate differentiation of samples, presenting results referring to each type of game.

Third, our sample is skewed towards male young people with higher education and is not representative of society as a whole. Future research needs to explore fan perceptions in a more culturally diverse environment and with a larger sample that contributes to the generalization of the results obtained.

Fourth, it is suggested in this study that the factors of the physical environment used for this eSports event are common to other types of eSports competition, similar to the research promoted by Jang et al. (17) in a sports context. However, the use of this instrument in other contexts must be cautious because the findings can change according to the arena (e.g., an open-air stadium), the eSports game-genre (e.g., an individual game vs. collective games), and the event location (e.g., survey application in other countries such as South Korea). Further studies should apply this research survey duly adapted to the sociocultural context of the event selected.

Fifth, this study followed the conceptual model proposed by Zhu et al. (2), using four dimensions to better reflect the eSports context. Although these dimensions help explain important aspects of the physical environment at eSports events, the model is still based on traditional sports and general service quality literature. This can limit its theoretical originality. Future research should explore additional dimensions that are more specific to eSports [e.g., virtual reality, screen integration or interactive technological features; (23)], using qualitative studies to generate context-specific constructs and expand the theoretical framework for assessing service quality.

## Data Availability

The original contributions presented in the study are included in the article/Supplementary Material, further inquiries can be directed to the corresponding author.

## References

[1] Funk D Pizzo A Baker B. Esport management: embracing eSport education and research opportunities. Sport Manag Rev. (2018) 21(1):7–13. 10.1016/j.smr.2017.07.008

[2] Zhu X Pyun D Manoli A. Developing a conceptual model of service quality for eSports. Quest. (2021) 73(4):375–90. 10.1080/00336297.2021.19766

[3] Statista. eSports market revenue worldwide from 2020 to 2025. (2023). Available at: https://www.statista.com/statistics/490522/global-eSports-market-revenue/ (Accessed March 15, 2023).

[4] Statista. eSports audience size worldwide from 2020 to 2025. (2023). Available at: https://www.statista.com/statistics/1109956/global-eSports-audience/ (Accessed March 15, 2023).

[5] Jenny S Manning R Keiper M Olrich T. Virtual (ly) athletes: where eSports fit within the definition of “Sport”. Quest. (2016) 69(1):1–18. 10.1080/00336297.2016.1144517

[6] Scholz T. (2019). eSports is Business: Management in the World of Competitive Gaming. Cham: Springer International Publishing. 10.1007/978-3-030-11199-1

[7] Jonasson K Thiborg J. Electronic sport and its impact on future sport. Sport Soc. (2010) 13(2):287–99. 10.1080/17430430903522996

[8] Pizzo A Baker B Na S Lee M Kim D Funk D. Esport vs sport: a comparison of spectator motives. Sport Mark Q. (2018) 27(2):108–23. 10.32731/smq.272.062018.04

[9] Hamari J Sjöblom M. What is eSports and why do people watch it? Internet Res. (2017) 27(2):211–32. 10.1108/intr-04-2016-0085

[10] Pu H Xiao S Kota RW. Virtual games meet physical playground: exploring and measuring motivations for live eSports event attendance. Sport Soc. (2021) 25(10):1886–908. 10.1080/17430437.2021.1890037

[11] Jang W Kim K Byon K. Social atmospherics, affective response, and behavioral intention associated with eSports events. Front Psychol. (2020) 11:1671. 10.3389/fpsyg.2020.01671 32849018 PMC7403203

[12] Seo Y. Electronic sports: a new marketing landscape of the experience economy. J Mark Manag. (2013) 29(13–14):1542–60. 10.1080/0267257X.2013.822906

[13] Taylor T. Raising the Stakes: E-Sports and the Professionalization of Computer Gaming. Cambridge, MA: MIT Press (2012).

[14] Ko Y Pastore D. Current issues and conceptualizations of service quality in the recreation sport industry. Sport Mark Q. (2004) 13(3):158–66.

[15] Theodorakis N Alexandris K Tsigilis N Karvounis S. Predicting spectators’ behavioural intentions in professional football: the role of satisfaction and service quality. Sport Manag Rev. (2013) 16(1):85–96. 10.1016/j.smr.2012.05.004

[16] Biscaia R. Revisiting the role of football spectators’ behavioral intentions and its antecedents. Open Sports Sci J. (2016) 9(1):3–12. 10.2174/1875399(01609010003

[17] Jang W Byon K Yim B. Sportscape, emotion, and behavioral intention: a case of the big four US-based major sport leagues. Eur Sport Manag Q. (2020) 20(3):321–43. 10.1080/16184742.2019.1607521

[18] Wakefield K Sloan H. The effects of team loyalty and selected stadium factors on spectator attendance. J Sport Manag. (1995) 9(2):153–72. 10.1123/jsm.9.2.153

[19] Wakefield K Blodgett J Sloan H. Measurement and management of the sportscape. J Sport Manag. (1996) 10(1):15–31. 10.1123/jsm.10.1.15

[20] Ko Y Zhang J Cattani K Pastore D. Assessment of event quality in major spectator sports. Manag Serv Qual. (2011) 21(3):304–22. 10.1108/09604521111127983

[21] Yoshida M James J. Service quality at sporting events: is aesthetic quality a missing dimension? Sport Manag Rev. (2011) 14(1):13–24. 10.1016/j.smr.2009.06.002

[22] Uhrich S Benkenstein M. Physical and social atmospheric effects in hedonic service consumption: customers’ roles at sporting events. Serv Ind J. (2012) 32(11):1741–57. 10.1080/02642069.2011.556190

[23] Jenny S Keiper M Taylor B Williams D Gawrysiak J Manning R Esports venues: a new sport business opportunity. J Appl Sport Manag. (2018) 10(1):8. 10.18666/JASM-2018-V10-I1-8469

[24] Calapez A Ribeiro T Almeida V Pedragosa V. Esports fan identity toward sponsor–sponsee relationship: an understanding of the role-based identity. Int J Sports Mark Spons. (2024) 25(1):42–66. 10.1108/IJSMS-02-2023-0030

[25] Foroughi B Shah K Ramayah T Iranmanesh M. The effects of peripheral service quality on spectators’ emotions and behavioural intentions. Int J Sports Mark Spons. (2019) 20(3):495–515. 10.1108/ijsms-08-2018-0082

[26] Chalip L. Towards social leverage of sport events. J Sport Tour. (2006) 11(2):109–27. 10.1080/14775080601155126

[27] Mehrabian A Russell J. An Approach to Environmental Psychology. Cambridge, MA: The MIT Press (1974).

[28] Hightower R Brady M Baker T. Investigating the role of the physical environment in hedonic service consumption: an exploratory study of sporting events. J Bus Res. (2002) 55(9):697–707. 10.1016/s0148-2963(00)00211-3

[29] Theodorakis ND Alexandris K. Can service quality predict spectators’ behavioral intentions in professional soccer? Manag Leis. (2008) 13(3):162–78. 10.1080/13606710802200852

[30] Leon M Hinojosa-Ramos M León-Lopez A Belli S López-Raventós C Florez H. Esports events trend: a promising opportunity for tourism offerings. Sustainability. (2022) 14(21):13803. 10.3390/su142113803

[31] Ribeiro T Calapez A Almeida V Matsuoka H. Understanding the role of sport values on social capital and word-of-mouth on the internet: a case study of esports games. Simul Gaming. (2023) 54(6):645–79. 10.1177/10468781231197175

[32] Qian T Wang J Zhang J Lu L. It is in the game: dimensions of eSports online spectator motivation and development of a scale. Eur Sport Manag Q. (2019) 20(4):458–79. 10.1080/16184742.2019.1630464

[33] Amor J Pérez-Campos C Molina-García N. Brand image in eSports events. Difference between players and non-players. J Sports Econ Manag. (2020) 10(2):102–13.

[34] Parasuraman A Zeithaml V Berry L. SERVQUAL: a multiple-item scale for measuring consumer perceptions of service quality. J Retail. (1988) 64(1):12–40.

[35] Foroughi B Shah K Nikbin D Hyun SS. The impact of event quality on fan satisfaction and game attendance in the context of professional soccer in Iran. Int J Sports Mark Spons. (2014) 15(3):40–56. 10.1108/IJSMS-15-03-2014-B005

[36] Wakefield K Blodgett J. The effect of the servicescape on customers’ behavioral intentions in leisure service settings. J Serv Mark. (1996) 10(6):45–61. 10.1108/08876049610148594

[37] Jung S Chen J Cai L. Beyond video game competition: novel dimensions of live ESports event experiences through co-creation. J Hospit Tourism Res. (2024) 48(8):1453–66. 10.1177/10963480231220282

[38] Bitner M. Servicescapes: the impact of physical surroundings on customers and employees. J Mark. (1992) 56(2):57–71. 10.1177/002224299205600205

[39] Wakefield K Blodgett J. Customer response to intangible and tangible servicefactors. Psychol Mark. (1999) 16(1):51–68. 10.1002/(SICI)1520-6793(199901)16:1<51::AID-MAR4>3.0.CO;2-0

[40] The Verge. Riot teamed Lil Nas X up with a holographic mech for 2022’s Worlds opening ceremony. (2022). Available at: https://www.theverge.com/2022/11/6/23440640/worlds-2022-opening-ceremony-league-of-legends-lil-nas-x (Accessed March 17, 2023).

[41] Kim S Yim B Byon K Yu J Lee S Park J. Spectator perception of service quality attributes associated with Shanghai formula one: importance and performance analysis approach. Int J Sports Mark Spons. (2016) 17(2):153–71. 10.1108/IJSMS-04-2016-011

[42] Newsubstance. League of Legends Worlds Final: Creating the set for a world-wide event. (2021). Available at: https://newsubstance.co.uk/case_study/league-of-legends-worlds-final/ (Accessed July 22, 2023).

[43] Accessibility. The state of accessibility in gaming in 2022. (2022). Available at: https://www.accessibility.com/blog/the-state-of-accessibility-in-gaming-in-2021 (Accessed July 20, 2023).

[44] Anderson J Gerbing D. Structural equation modeling in practice: a review and recommended two-step approach. Psychol Bull. (1988) 103(3):411–23. 10.1037/0033-2909.103.3.411

[45] Ryu K Jang S. The effect of environmental perceptions on behavioral intentions through emotions: the case of upscale restaurants. J Hospit Tourism Res. (2007) 31(1):56–72. 10.1177/1096348006295506

[46] Jeong Y Kim E Kim S. Understanding active sport tourist behaviors in small-scale sports events: stimulus-organism-response approach. Sustainability. (2020) 12(19):8192. 10.3390/su12198192

[47] Inygon. Portfólio. (2023). Available at: https://inygon.com/portfolio (Accessed April 12, 2023).

[48] Feira Internacional de Lisboa. Sobre a FIL. (2023). Available at: https://www.fil.pt/sobre-a-fil/ (Accessed April 08, 2023).

[49] Inygon. About us. (2023). Available at: https://www.inygon.com/about (Accessed April 12, 2023).

[50] Childress R Crompton J. A comparison of alternative direct and discrepancy approaches to measuring quality of performance at a festival. J Travel Res. (1997) 36(2):43–57. 10.1177/004728759703600207

[51] Tsuji Y Bennett G Zhang J. Consumer satisfaction with an action sports event. Sport Mark Q. (2007) 16(4):199–208.

[52] Banville D Desrosiers P Genet-Volet Y. Translating questionnaires and inventories using a cross-cultural translation technique. J Teach Phys Educ. (2000) 19(3):374–38. 10.1123/jtpe.19.3.374

[53] Pollit D Beck C. The content validity index: are you sure you know what’s being reported? Critique and recommendations. Res Nurs Health. (2006) 29(5):489–97. 10.1002/nur.20147 16977646

[54] Marôco J. Análise Estatística com o SPSS Statistics.: 7ª edição. ReportNumber, Lda (2018).

[55] Hair J Black W Babin B Anderson R Tatham R. Multivariate data analysis. New Jersey: Prentice Hall (1988). Vol. 5, No. 3, p. 207–219.

[56] Fornell C Larcker D. Evaluating structural equation models with unobservable variables and measurement error. J Mark Res. (1981) 18(1):39–50. 10.1177/002224378101800104

[57] Hair JF Ringle CM Sarstedt M. PLS-SEM: indeed a silver bullet. J Mark Theory Pract. (2011) 19(2):139–52. 10.2753/mtp1069-6679190202

[58] Nunnally JC Bernstein I. Psychometric Theory (3rd ed.). New York: McGraw Hill (1994).

[59] Biscaia R Correia A Yoshida M Rosado A Marôco J. The role of service quality and ticket pricing on satisfaction and behavioural intention within professional football. Int J Sports Mark Spons. (2013) 14(4):301–25. 10.1108/ijsms-14-04-2013-b004

[60] Lisbon International Fair. About FIL. (2023). Available at: https://www.fil.pt/sobre-a-fil/ (Accessed February 04, 2023).

[61] Kline RB. Principles and Practice of Structural Equation Modeling (2nd ed.). New York: Guilford (2005).

