# Supplementary material for: Hosting eSports events: the quality-response-behavioural intentions relationship of eSports fans
Source: Front Sports Act Living. 2025 Jun 9;7:1547097. doi: 10.3389/fspor.2025.1547097 (PMC12183061; doi:10.3389/fspor.2025.1547097)
Supplement: Supplementary file 1 [file Datasheet1.docx]

Supplementary Material

## *Appendix – CFA item statistics*

| **Variables** | **Scores** | | |
| --- | --- | --- | --- |
|  | M (SD) | Factor loadings | CR |
| ***Atmosphere*** | 5.67(.95) |  | .73 |
| 1.The eSports arena has a pleasant scent. |  | .705 |  |
| 2.The light scheme is excellent at the eSports arena. |  | .816 |  |
| 3.The background music the eSports arena is good. |  | .545 |  |
| ***Equipment*** | 6.37(.77) |  | .83 |
| 4.The giant screens are pleasant to watch. |  | .796 |  |
| 5.The eSports arena has high quality giant screens |  | .784 |  |
| 6.The eSports arena has a high-quality sound system. |  | .796 |  |
| ***Facility Design*** | 5.43(1.12) |  | .85 |
| 7.This eSports arena architecture makes it attractive. |  | .718 |  |
| 8.This eSports arena is decorated based on an appealing theme. |  | .801 |  |
| 9.This eSports arena walkways are wide enough to handle the crowds. |  | .896 |  |
| ***Accessibility*** | 4.77(1.31) |  | .83 |
| 10.Signs at this eSports arena help you know where you are going. |  | .559 |  |
| 11.Signs at this eSports arena gives clear directions of where things are located. |  | .883 |  |
| 12.At this eSports arena there is a good accessibility for those with special needs |  | .877 |  |
| ***Affective Responses*** | 5.63(1.11) |  | .89 |
| 13. Happy-Unhappy |  | .819 |  |
| 14. Annoyed-Pleased |  | .900 |  |
| 15. Disappointed-Delighted |  | .828 |  |
| ***Revisit Intention*** | 5.38(1.48) |  | .91 |
| 16.I plan to continue attending my favourite eSports game’s events frequently. |  | .947 |  |
| 17.I intend to attend my favourite esports game’s event soon. |  | .935 |  |
| 18.I will attend the next edition of this event. |  | .743 |  |
| ***Word-of-mouth*** | 4.98(1.66) |  | .87 |
| 19.I will recommend a visit to this event to at least one of my family members. |  | .841 |  |
| 20.I will recommend a visit to this event to people who are generally not interested in eSports. |  | .796 |  |
| 21. I will recommend a visit to this event to friends and acquaintances. |  | .843 |  |

Source: Authors own creation.
